# Longitudinal evaluation of external quality assessment results for CA 15-3, CA 19-9, and CA 125

**DOI:** 10.3389/fmolb.2024.1401619

**Published:** 2024-06-20

**Authors:** Marcel Kremser, Nathalie Weiss, Anne Kaufmann-Stoeck, Laura Vierbaum, Arthur Schmitz, Ingo Schellenberg, Stefan Holdenrieder

**Affiliations:** ^1^ INSTAND e.V., Society for Promoting Quality Assurance in Medical Laboratories, Duesseldorf, Germany; ^2^ Center of Life Sciences, Institute of Bioanalytical Sciences (IBAS), Anhalt University of Applied Sciences, Bernburg, Germany; ^3^ Munich Biomarker Research Center, Institute of Laboratory Medicine, Deutsches Herzzentrum München, Technische Universität München, Munich, Germany

**Keywords:** external quality assessment, tumor marker, cancer antigen, CA 15-3, CA 19-9, CA 125, EQA, INSTAND

## Abstract

**Background:**

Tumor markers are established laboratory tools that help to diagnose, estimate prognosis, and monitor the course of cancer. For meaningful decision-making in patient care, it is essential that methods and analytical platforms demonstrate high sensitivity, specificity, precision, and comparability. Regular participation at external quality assessment (EQA) schemes is mandatory for laboratories. Here, a longitudinal evaluation of EQA data was performed to assess the performance of tumor marker assays over time.

**Methods:**

Longitudinal data of the cancer antigens (CA) 15-3 (n = 5,492), CA 19-9 (n = 6,802), and CA 125 (n = 5,362) from 14 INSTAND EQAs conducted between 2019 and 2023 were evaluated. A median of 197, 244 and 191 laboratories participated at the EQAs for CA 15-3, CA 19-9 and CA 125, respectively. Data evaluation encompasses intra- and inter-manufacturer specific variations over time, assay precision, and adherence to the EQA limits of ±24% for CA 15-3, ±27% for CA 19–9 and ±36% for CA 125.

**Results:**

The study showed median manufacturer-dependent differences of up to 107% for CA 15-3, 99% for CA 125, and even 549% for CA 19-9 between the highest and the lowest methods over the studied period. Regarding the normalized median of all methods, the values of the most deviant methods were 0.42 for CA 15-3, 7.61 for CA 19-9, and 1.82 for CA 125. Intra-manufacturer variability was generally low, with median coefficients of variation (CV) below 10%. As the methods were evaluated according to method-specific consensus values, most participants passed the EQAs within the acceptance criteria. When the criteria were consistently set at 24%, the central 90% of participants passed the EQAs in 78.6%–100% for CA 15-3 (with exception of AX), 89.3%–100% for CA 125, and 64.3%–100% for CA 19-9.

**Conclusion:**

While intra-method precision of most analytical platforms is acceptable for all three tumor markers, considerable inter-method variability was observed over the whole studied period demonstrating the necessity for better standardization and harmonization of the methods, development of international reference materials, and comprehensive commutability studies with patient samples.

## 1 Introduction

Cancer remains a challenge to public health worldwide (Bray et al., 2021; Sung et al., 2021). As our understanding of cancer biology continues to advance, so does the need for improved diagnostic tools for the detection, risk assessment, and monitoring of therapeutic responses. Tumor markers have risen in prominence as potential indicators for the presence and progression of cancer (Filella et al., 2023).

Among the diverse array of tumor markers, the cancer antigens (CA) 15-3, CA 19-9, and CA 125 have emerged as useful tools in the detection and management of various cancer entities (Stieber and Heinemann, 2008a). CA 15-3, also known as Mucin-1 (MUC-1), is a 300 kDa carbohydrate antigen found in normal breast and breast cancer cells (Gang et al., 1985; Duffy et al., 2000). It is also expressed by other types of cancer, such as lung cancer and gastric cancer, and appears in elevated levels in the blood serum and plasma of patients with non-cancer-related conditions like cirrhosis, hepatitis and benign breast diseases (Duffy et al., 2010).

CA 19-9, also known as Sialyl Lewis-antigen, is a 36 kDa glycolipid that emerges from the generation of a monoclonal antibody against a colon carcinoma cell line (DelVillano and Zurawski, 1983). Elevated levels of CA 19-9 are notably exhibited in the blood of patients with various malignancies, including gastric, lung, colon and pancreatic cancers (Lee et al., 2020). Furthermore, high levels of CA 19-9 in the blood are observed in non-malignant conditions such as benign pancreatobiliary, hepatic and pulmonary diseases, and in cases of thyroiditis, diabetes mellitus, and autoimmune disorders (Trape et al., 2011; Kim et al., 2020).

CA 125, also known as MUC-16, is a 200 kDa membrane glycoprotein expressed on the surface of ovarian cancer cells (Charkhchi et al., 2020). It is defined by the monoclonal antibody OC125, which is derived from human ovarian cancer cell lines. In addition to ovarian cancer, elevated levels in the blood are found in conjunction with lung, endometrial, pancreatic, breast, and colon cancer, as well as with physiological conditions such as menstruation and pregnancy (Ghosh et al., 2013). Given its susceptibility of being elevated under a range of circumstances, CA 125 is used in combination with other tumor markers, like human epididymis protein 4 (HE4), to assess the risk of suspicious pelvic masses (Moore et al., 2009; Escudero et al., 2011).

Although extensive research has been conducted on these tumor markers, challenges persist in achieving standardization and harmonization across methods (Mongia et al., 2006; Slev et al., 2006; La’ulu and Roberts, 2007; Passerini et al., 2007; Serdarevic, 2018). In 2005, the Society for Promoting Quality Assurance in Medical Laboratories (INSTAND) observed a manufacturer-dependent bias of up to 44% for CA 15-3, 194% for CA 19-9 and 162% for CA 125 as part of external quality assessment (EQA) results (Reinauer and Wood, 2005). INSTAND is accredited according to ISO17043 and is a reference institute of the German Medical Association. It has been conducting EQAs since 1966. Considerable variation has also been reported in clinical studies that compare different manufacturers (Stieber et al., 2008b; Holdenrieder et al., 2008; Molina et al., 2008). This is a matter of concern, as the ability to compare results across laboratories, manufacturers, and platforms is crucial for the meaningful interpretation of clinical data. This is particularly true given that the reference limits of different methods are often similar (La’ulu and Roberts, 2007). If cancer patients undergoing therapy or post-treatment surveillance receive tumor marker results from different laboratories utilizing different methods, the lack of standardization and harmonization can lead to erroneous interpretations of the marker dynamics. Furthermore, EQA providers are required to establish acceptance criteria for method-specific EQA schemes, which are essential for the interpretation of clinically meaningful results. Additionally, they must monitor the performance of analytes and methods over time.

In a recent analysis of EQA data on the current quality of the tumor markers alpha-feto protein (AFP) and carcinoembryonic antigen (CEA), for which there are international reference standard materials, we found a better level of standardization between 2018 and 2022 compared to that reported in 2005 (Wojtalewicz et al., 2023). In this study we performed a longitudinal assessment of EQA data for the tumor markers CA 15-3, CA 19-9, and CA 125 for which international reference standard materials have not yet been developed. We compared intra- and inter-method variations between EQA participants using the most common analytical platforms and tested their adherence to EQA limits.

## 2 Materials and methods

### 2.1 Sample materials

The matrix for the EQA samples was composed of human serum pools stabilized with 0.02% sodium azide. No other synthetic substances were added. To reach defined tumor marker concentrations, the matrix was spiked with non-synthetic tumor antigens from respective tumor tissue cell lines. Sample concentrations were selected based on clinical relevance and in accordance with the guidelines of the German Medical Association (RiliBÄK). The manufacturer declared and confirmed the homogeneity and stability of each sample batch. During the EQA surveys, the liquid samples were stored at 2°C–8°C until shipment.

### 2.2 EQA procedure

The INSTAND EQA scheme for tumor marker detection is conducted six times a year on a global scale. There are no exclusions for participants. For each survey, participating laboratories receive two EQA samples with different concentrations. The laboratories are required to report their quantitative results for CA 15-3, CA 19-9 and CA 125, along with other tumor markers, and provide information to INSTAND about the respective analytical platforms, methods, reagents, and manufacturers. Participating laboratories report this information via the RV-Online platform (https://rv-online.instandev.de).

As there is no available reference method for tumor marker quantification, the consensus value of manufacturer-specific collectives, calculated using algorithm A (ISO13528, (2022), Section C3), serves as the target value for evaluating participant results and laboratory certification. The EQA passing criterion for CA 15-3 is set at ±24% of the consensus value over the entire evaluation period. This is in accordance with the RiliBÄK (Bundesärztekammer, 2023). For CA 19-9 and CA 125, which are not covered in the current guideline, the EQA criteria are set at ±27% and ±36%, respectively.

### 2.3 Data analysis and statistics

In the present study EQA surveys conducted between January 2019 and May 2023 for the tumor markers CA 15-3, CA 19-9 and CA 125 were examined. As in the previously published tumor marker study (Wojtalewicz et al., 2023), only data from the three annual EQAs with the highest number of participants, namely January, May, and October, were included in the evaluation (Supplementary Table S1). The lower participant number EQA schemes have been excluded due to low statistically significance. In total 14 CA 15-3, CA 19-9 and CA 125 EQA surveys with two samples each were analyzed.

The EQA samples had different concentrations of the tumor markers that mirrored the relevant value range for clinical decision making. This was close to the cut-off values of the so-called reference range (95^th^ percentile of healthy individuals), which is around 30 kU/L for CA 15-3, 35 kU/L for CA 125, and 37 kU/L for CA 19-9 for most manufacturers and methods, and at slightly or strongly elevated levels as often seen in different cancer stages. For better orientation, cut-off values are highlighted with a red line in the figures.

The EQA data were analyzed in a manufacturer-dependent manner (Supplementary Table S2). We focused on manufacturer collectives with a minimum of six participants per survey, resulting in six collectives for the analysis of the CA 15-3 results, seven collectives for CA 19-9, and six collectives for CA 125. These were, in alphabetical order, Abbott (AB), Beckman (BE), bioMérieux (AX), Diasorin (DO), Roche (RO), Siemens (SI), and Tosoh (TH, for CA 19-9 only).

The results were illustrated using combined dot plots and box plot diagrams to visualize the distributions of the values in terms of median, interquartile range, and whiskers and to make them comparable over time.

The SI collective comprised four manufacturer sub-collectives consolidated under Siemens. In some EQA surveys, we observed a multimodality in the SI collective, but to gain a comprehensive understanding of the value distribution, all results from the SI cohort were included in the general box plot analysis. Additionally, the SI collective was divided into subgroups (Supplementary Table S1; Supplementary Figures S1–S4). Due to the multimodality of the SI collective, we specifically presented the normalized median for the more substantial sub-collectives Bayer Health (BG), DPC Biermann (DG) and Siemens Healthineers (SIE).

The collective median of each survey was normalized in relation to the overall median of the respective survey. The coefficients of variation (CVs) were calculated to assess the scatter within the manufacturer collectives; for the SI collective, the three sub-collectives BG, DG and SIE were considered separately.

In a further step, the inter-laboratory performance quality of CA 15-3, CA 19-9 and CA 125 as well as the manufacturer-dependent value distribution were analyzed in relation to the EQA success criteria. Here the central 80% (10th to 90th percentiles) and the central 90% (5th to 95th percentiles) of the participants of each manufacturer were compared to the acceptance criteria of each tumor marker.

We used jmp 17.2.0 from SAS Institute (Cary, NC, United States) for the basic statistical analyses. The overlay images were generated using version 2.10.34 of the Gnu image manipulation software.

## 3 Results

The data from the 14 EQA surveys, conducted in January, May and October between 2019 and 2023, were examined for the tumor markers CA 15-3, CA 19-9, and CA 125. The participating laboratories collectively provided 5,492 results for CA 15-3, 6,802 results for CA 19-9 and 5,362 results for CA 125. A median of 197 laboratories participated at the EQAs for CA 15-3 (minimum 172, maximum 219), 244 laboratories for CA 19-9 (minimum 214, maximum 275), and 191 laboratories for CA 125 (minimum 165, maximum 220). The detailed numbers of results per manufacturer are displayed in Supplementary Table S2. Regarding outlier management, sample mix-ups or reporting errors resulted in the exclusion of 35 results for CA 15-3, 20 results for CA 19-9, and 16 results for CA 125.

### 3.1 CA 15-3 EQA results

**FIGURE 1:**
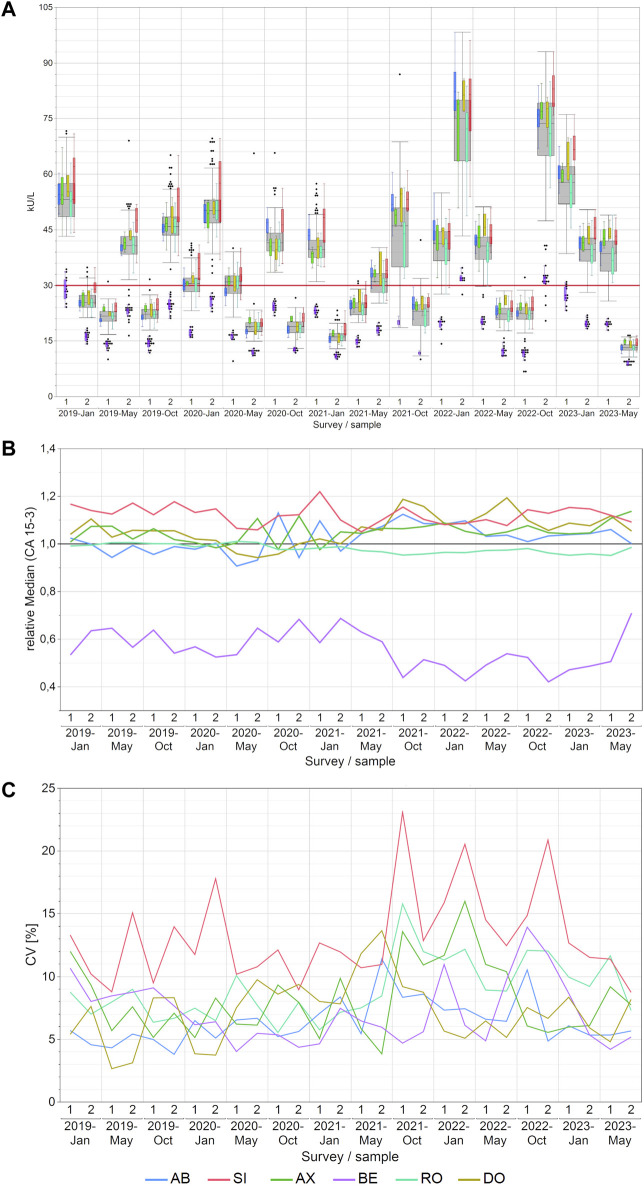
Manufacturer-dependent analysis of CA 15-3 EQA results, encompassing an all-results overview **(A)**, comparisons of manufacturer-dependent median differences relative to the overall median **(B)**, and evaluations of manufacturer-dependent CVs **(C)** between 2019 and 2023. Data are presented for two samples per survey. The gray boxes represent all results for the respective sample, while smaller, colored box plots overlay the total results (blue: AB, green: AX, cyan: RO, violet: BE, red: SI, ochre: DO). A red line marks the 30 kU/L cut-off value, and black dots denote outliers excluded from the colored boxes. The whiskers extend from the 1st quartile minus 1.5 times the interquartile range to the 3rd quartile plus 1.5 times the interquartile range.

Notable disparities in concentrations of CA 15-3 were observed across manufacturers, with median variations reaching as high as 107% between BE and SI and the maximum variations reaching as high as 171% between BE and DO. For other methods, the differences were lower as displayed in detail in Supplementary Table S3. The BE collective consistently reported the lowest values and never overlapped with results from other collectives (Figure 1A). In contrast, the SI collective often reported the highest values. Excluding the BE collective from the analysis substantially reduced the highest manufacturer-specific concentration differences to 25%, as seen between SI and AX in the January 2021 survey.

The trend of BE consistently reporting the lowest values became even more apparent when the normalized median differences between manufacturer collectives (Figure 1B) and the median values along with the minimum and maximum values of the normalized median differences for each manufacturer collective were considered (Supplementary Table S4). Notably, the BE collective exhibited the lowest relative median value of 0.54—noticeably lower than the other collectives.

The median intra-manufacturer coefficients of variation (CVs) for CA 15-3 measurements mostly remained below 10% (maximum 16%), pointing to a high level of assay precision (Figure 1C; Supplementary Table S5). The only exception to this pattern was the SI collective, which achieved a maximum CV of up to 23%. Subdividing the SI collective into sub-collectives showed lower median CV below 10% (maximum 20% for the SIE subgroup; Supplementary Figure S1A).

### 3.2 CA 19-9 EQA results

**FIGURE 2:**
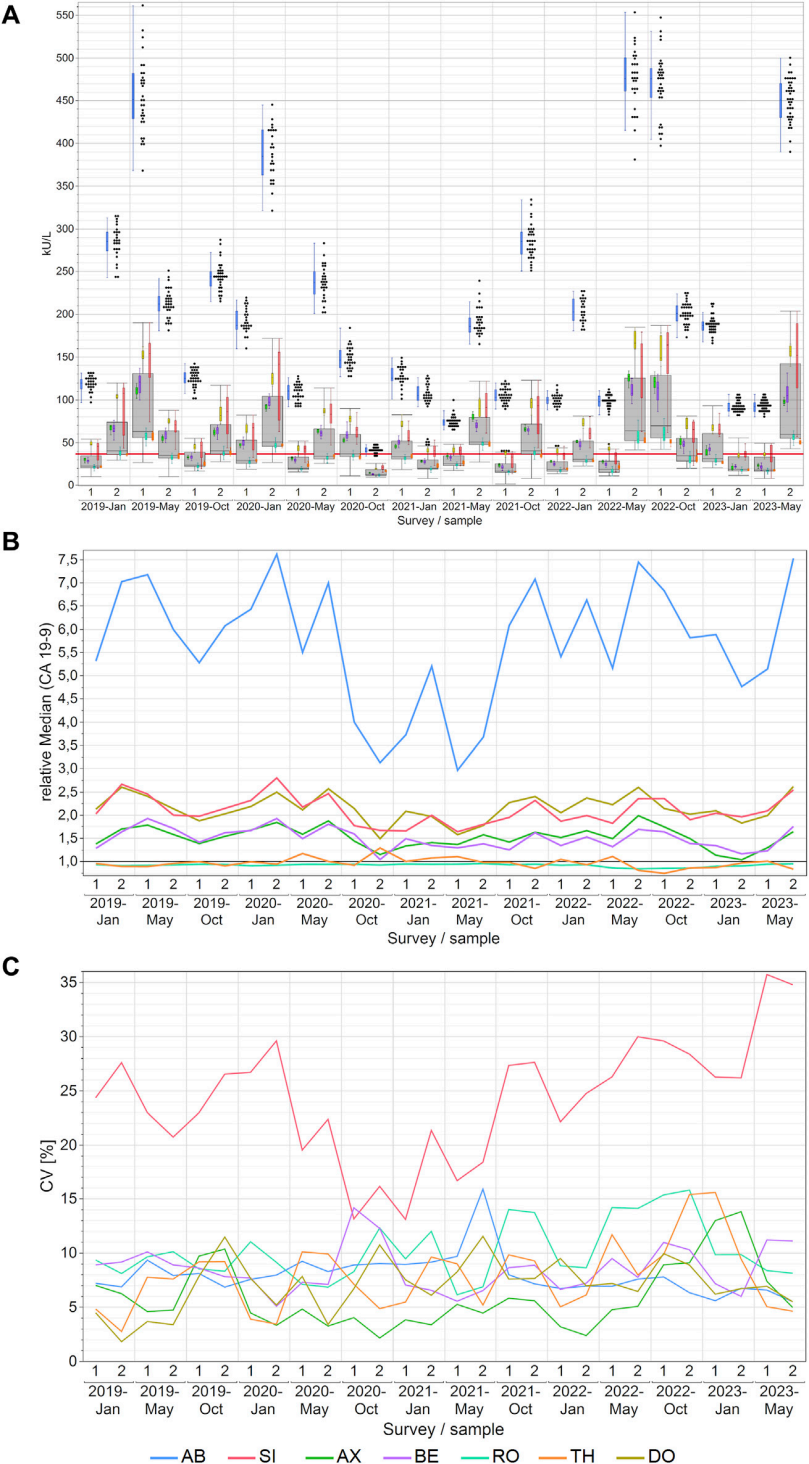
Manufacturer-dependent analysis of CA 19-9 EQA results, encompassing an all-results overview **(A)**, comparisons of manufacturer-dependent median differences relative to the overall median **(B)**, and evaluations of manufacturer-dependent CVs **(C)** between 2019 and 2023. Data are presented for two samples per survey. The gray boxes represent all results for the respective sample, while smaller, colored box plots overlay the total results (blue: AB, green: AX, cyan: RO, violet: BE, red: SI, ochre: DO, orange: TH). A red line marks the 37 kU/L cut-off value, and black dots denote outliers excluded from the colored boxes. The whiskers extend from the 1st quartile minus 1.5 times the interquartile range to the 3rd quartile plus 1.5 times the interquartile range.

For CA 19-9, the AB collective consistently reported considerably higher values and never overlapped with the other collectives. Its values occasionally reached very high levels of approximately 560 kU/L. This contrasted starkly with other companies, where measurements typically did not exceed 200 kU/L. Conversely, the RO and TH collectives consistently reported the lowest values for CA 19-9 (Figure 2A). Median variations across manufacturers reached as high as 549% between AB and RO and the maximum variations reaching as high as 822% between AB and TH in May 2022. For other methods, the differences were lower as displayed in detail in Supplementary Table S3.

Similarly, these trends are even more evident in the relative collective medians of CA 19-9 when normalized to the overall median of the sample results (Figure 2B). Excluding the AB collective from the analysis substantially reduced the maximum manufacturer-specific differences to 222% when the DO collective, which had the highest value, is compared with the TH collective, which had the lowest value in May 2022. Notably, the AB collective exhibited the highest maximum normalized median difference of 7.61 and a median normalized median of 5.84, indicative of its substantial deviation from the overall median. Conversely, the medians for RO and TH for the normalized median were 0.93 and 0.96 respectively, and the TH collective displayed the lowest maximum normalized median difference of 1.29 (Supplementary Table S6).

The variation within individual collectives was, in fact, quiet low, with median CVs mostly below 10% (maximum CV 16%). This indicates a commendable level of assay precision (Figure 2C; Supplementary Table S5). Nevertheless, it should be noted that the SI collective sometimes displayed CVs as high as 36%. Dividing the SI collective into sub-collectives meant that the resulting subgroups, although still occasionally displaying CVs as high as 35% as in the case of the DG collective in January 2022 (Supplementary Figure S1B), had median CVs between 10% and 12% which is comparable to the other manufacturer collectives (Supplementary Table S7).

### 3.3 CA 125 EQA results

**FIGURE 3:**
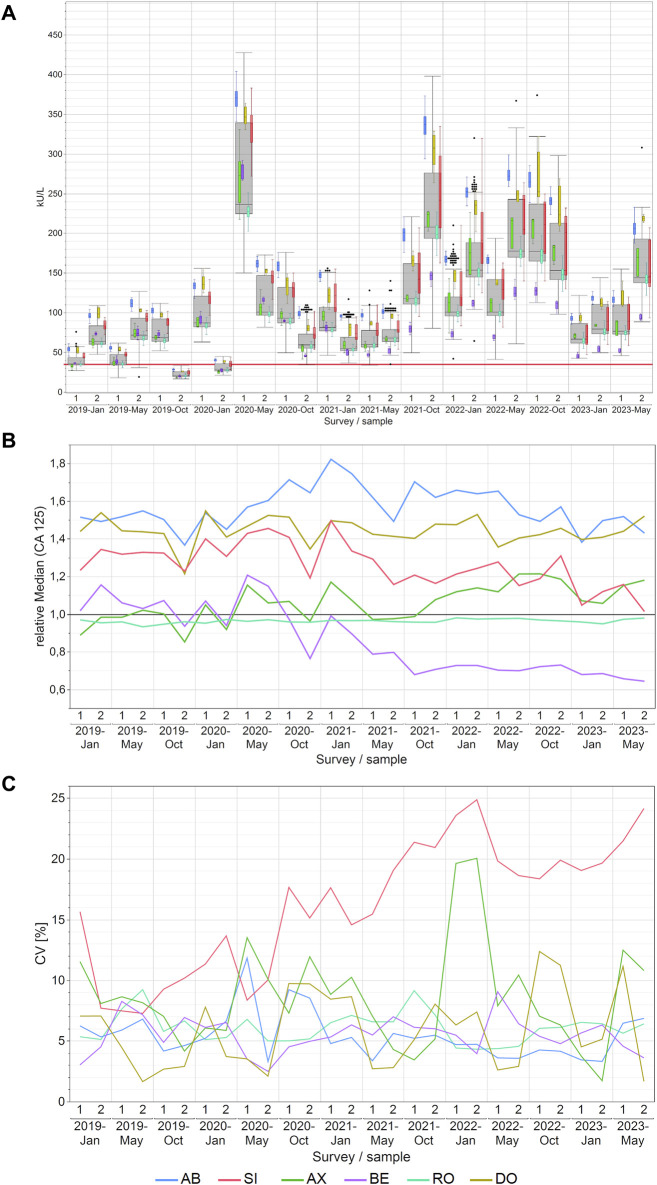
Manufacturer-dependent analysis of CA 125 EQA results, encompassing an all-results overview **(A)**, comparisons of manufacturer-dependent median differences relative to the overall median **(B)**, and evaluations of manufacturer-dependent CVs **(C)** between 2019 and 2023. Data are presented for two samples per survey. The gray boxes represent all results for the respective sample, while smaller, colored box plots overlay the total results (blue: AB, green: AX, cyan: RO, violet: BE, red: SI, ochre: DO). A red line marks the 35 kU/L cut-off value, and black dots denote outliers excluded from the colored boxes. The whiskers extend from the 1st quartile minus 1.5 times the interquartile range to the 3rd quartile plus 1.5 times the interquartile range.

In the case of CA 125, either the AB or DO collective consistently reported the highest measured values for each EQA survey. A noteworthy change was observed in the performance of the BE collective, which consistently remained in the interquartile range of the overall box plot (grey box) before 2021, and then its values were only in the lower whisker range of all results (Figure 3A). Notable disparities in concentrations of CA 125 were observed across manufacturers, with median variations reaching as high as 99% between AB and BE and the maximum variations reaching as high as 151% between AB and BE in October 2021. For other methods, the differences were lower as displayed in detail in Supplementary Table S3.

In contrast to −20% to +20% before October 2021, the normalized median values of the BE collective from October 2021 onwards maintained a very consistent value for CA 125 measurements, with a bias of −30% in comparison to the overall median (Figure 3B). The AB collective had the highest normalized median value of 1.54, while the AX and RO collectives exhibited lower median values of 1.06 and 0.96 respectively (Supplementary Table S8).

Regarding method variability, the SI collective notably exhibited the highest scatter of results among the manufacturer collectives, with median CVs reaching 18% (maximum 25%). In contrast, the other collectives consistently maintained median CVs between 5% and 8% (maximum 20%; Figure 3C). As for the other CA markers studied in this paper, the high CVs of the SI collective went down once it was divided into its sub-collectives (Supplementary Figure S1C). The median CVs were 8% for BG, 6% for DG and 11% for SIE. Thus, they are more comparable to the median CVs of the other manufacturer collectives, which ranged from 5% to 8%, than to the overall SI collective with a median CV of 18% (Supplementary Table S9).

### 3.4 Evaluation of EQA results with respect to the current assessment limits

**FIGURE 4:**
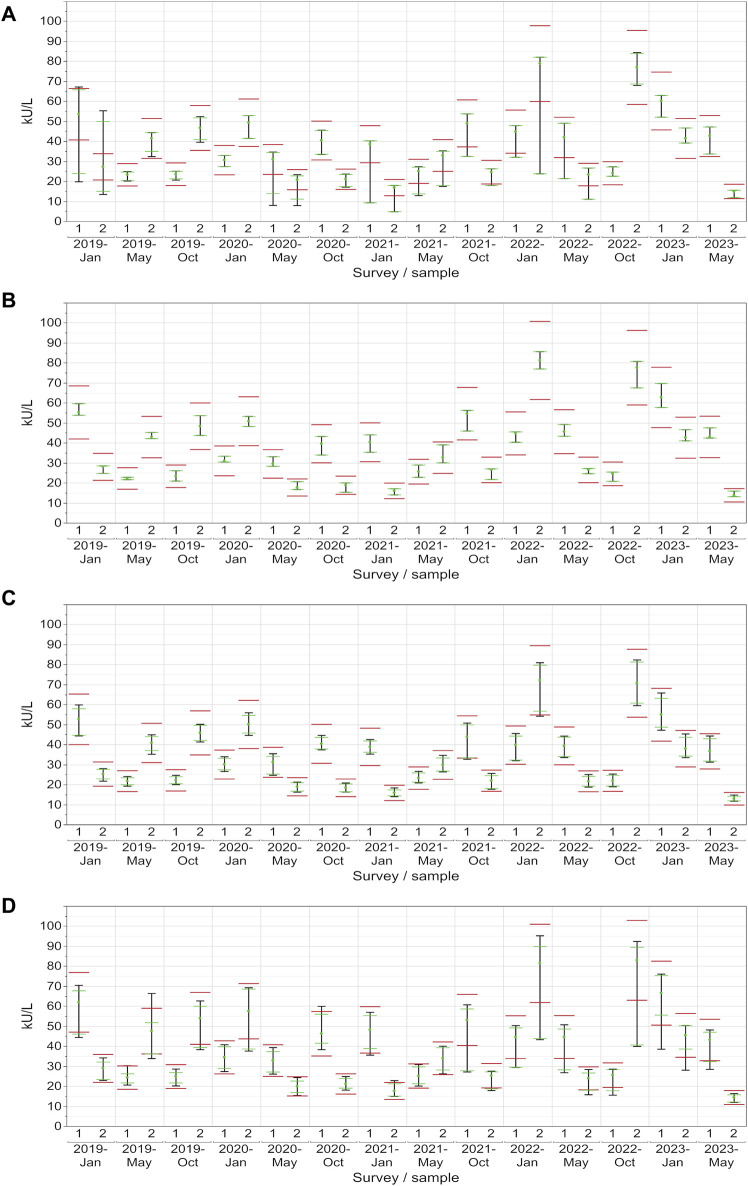
Manufacturer-specific evaluation of EQA results for CA 15-3 with respect to the current assessment limits for the AX **(A)**, DO **(B)**, RO **(C)** and SI **(D)** collectives. The green dot represents the median of all results within each respective collective and EQA survey. Assessment limits of ±24% are highlighted with red lines, while green lines indicate the median for 80% of the results, and a black line signifies the median for 90% of the results.

For CA 15-3, the AX collective displayed more variability, with the central 90% exceeding the limits in approximately half of the samples (Figure 4A). In contrast, the central 90% of values from the DO collective consistently adhered to the assessment limits for each sample (100% passing rate: ±24%), thereby demonstrating excellent performance (Figure 4B). The RO collective’s results closely mirrored those of the DO collective, with only a minor deviation occurring twice when the central 90% were not able to pass the lower assessment limit (92% passing rate) (Figure 4C). The SI collective consistently exceeded the assessment limits in over half of the instances and the central 90% of SI passed the assessment limits only nine times (Figure 4D). Both collectives displayed fluctuations above and below the threshold. When evaluated separately, the three SI subtypes (BG, DG, SIE) had passing rates of 93%–96% (Table 1).

**TABLE 1:** Evaluation of EQA results with respect to the current assessment limits and stricter, uniform assessment limits of 24%.

	CA 15-3 (±24%)	CA 19-9 (±27%)	CA 19-9 (±24%)	CA 125 (±36%)	CA 125 (±24%)
Manufacturer	passing (5%–95%) [n/%]	passing (5%–95%) [n/%]	passing (5%–95%) [n/%]	passing (5%–95%) [n/%]	passing (5%–95%) [n/%]
AB	24/85.7	27/96.4	26/92.9	27/96.4	25/89.3
AX	14/50.0	24/85.7	24/85.7	26/92.9	22/78.6
BE	22/78.6	26/92.9	23/82.1	28/100.0	28/100.0
DO	28/100.0	28/100.0	28/100	28/100.0	26/92.9
RO	26/92.9	27/96.4	24/85.7	28/100.0	28/100.0
SI	9/32.1	1/3.6	1/3.6	18/64.3	8/28.6
SI—BG	26/92.9	23/82.1	18/64.3	25/89.3	18/64.3
SI—DG	26/92.9	21/75	18/64.3	28/100.0	26/92.9
SI—SIE	26/96.4	19/67.9	19/67.9	25/89.3	19/67.9
TH		24/85.7	22/78.6		

**FIGURE 5:**
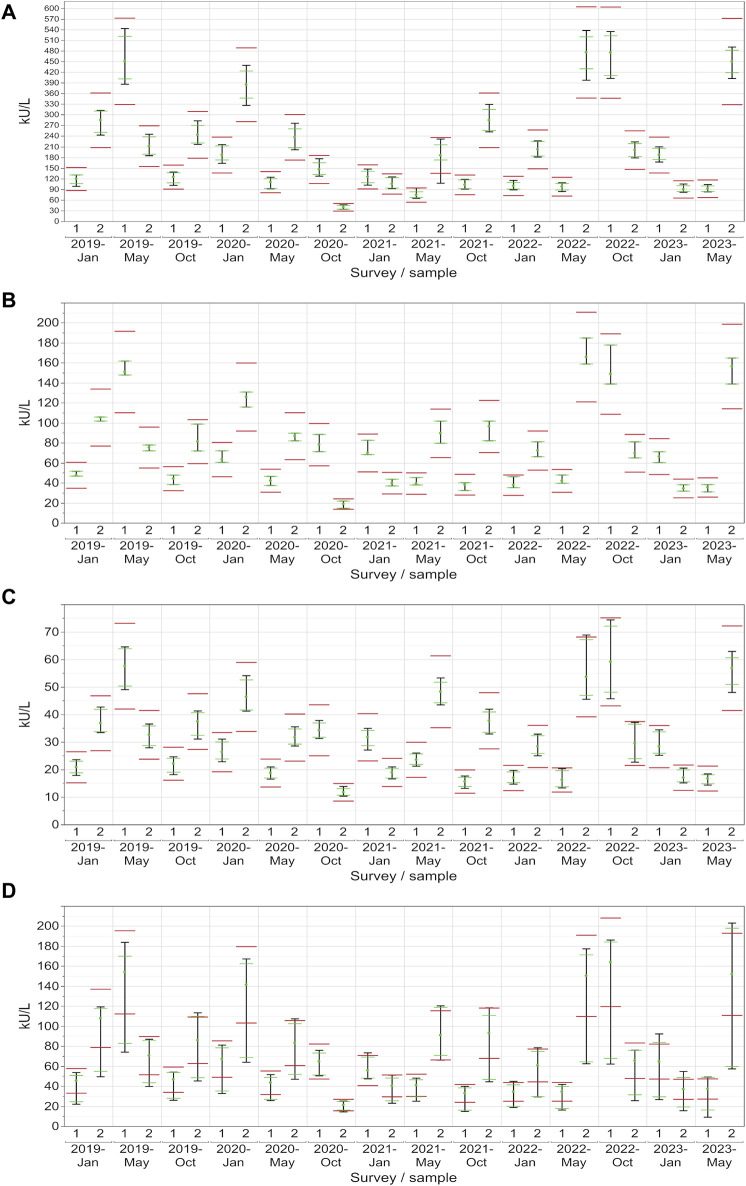
Manufacturer-specific evaluation of EQA results for CA 19-9 with respect to the current assessment limits for the AB **(A)**, DO **(B)**, RO **(C)** and SI **(D)** collectives. The green dot represents the median of all results within each respective collective and EQA survey. Assessment limits of ±27% are highlighted with red lines, while green lines indicate the median for 80% of the results, and a black line signifies the median for 90% of the results.

In the case of CA 19-9, the AB collective demonstrated exceptional consistency, with the central 90% not passing the lower assessment limit in just one instance (96% passing rate) (Figure 5A). On the other hand, the central 90% of values from the DO collective consistently remained within the assessment limits for each sample (100% passing rate: ±27%), demonstrating a robust performance (Figure 5B). Similar to CA 15-3, the RO collective delivered commendable results, with only one instance with the central 90% of laboratories being outside the upper assessment limit (96% passing rate) (Figure 5C). Notably, the SI collective consistently exceeded the assessment limit in every instance except once in October 2020 (Figure 5D). When evaluated separately, the three SI subtypes (BG, DG, SIE) had passing rates of 68%–82% (Table 1).

**FIGURE 6:**
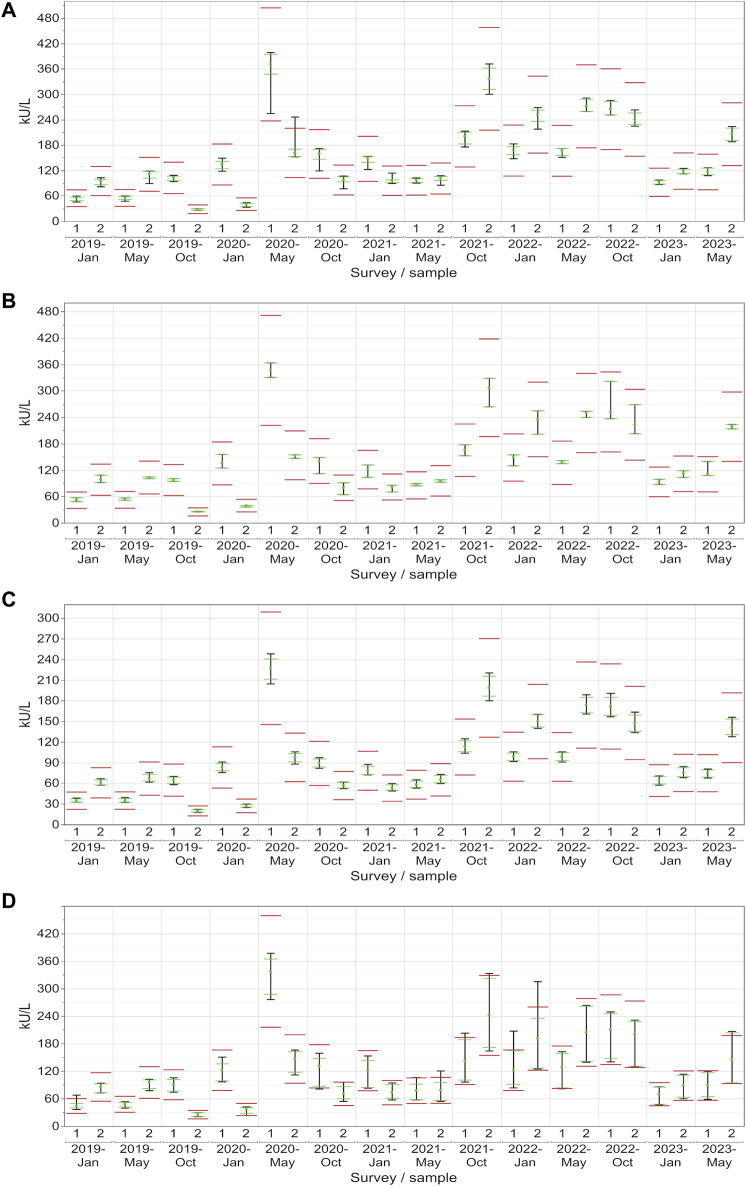
Manufacturer-specific evaluation of EQA results for CA 125 with respect to the current assessment limits for the AB **(A)**, DO **(B)**, RO **(C)** and SI **(D)** collectives. The green dot represents the median of all results within each respective collective and EQA survey. Assessment limits of ±36% are highlighted with red lines, while green lines indicate the median for 80% of the results, and a black line signifies the median for 90% of the results.

When looking at CA 125, the AB collective also performed comparably well, with only a single instance where the central 90% slightly did not pass the upper assessment limit (96% passing rate) (Figure 6A). Both the RO and DO collectives consistently maintained all values within the assessment limits for each sample (100% passing rate: ±36%), which reflects a strong performance (Figures 6B, C). Even the central 90% of the more variable SI collective exceeded the assessment limit on only 10 out of 28 occasions (Figure 6D). When evaluated separately, the three SI subtypes (BG, DG, SIE) had passing rates of 89%–100% (Table 1).

When the assessment limits were adjusted so that each tumor marker had the 24% passing limit as stipulated for CA 15-3 by the RiliBÄK guidelines (Bundesärztekammer, 2023), the central 90% of most collectives would still pass on many occasions (Table 1; Supplementary Figures S5, S6) with passing rates of 79%–100% for most manufacturers for CA 19-9 (only the 3 SI subtypes remained below 70%) and 79%–100% for most manufacturers for CA 125 (only 2 of the SI subtypes remained below 70%).

## 4 Discussion

The utilization of EQA material for comparative analysis provides a standardized framework for evaluating laboratory performance across different assays. While some EQA institutions in other countries use patient samples, similar phenomena and variations are observed for both materials (van Rossum et al., 2024). This study undertakes a thorough re-evaluation of recent EQA data spanning from 2019 to 2023 for the biomarkers CA 15-3, CA 19-9 and CA 125 and highlights notable variations in the performance of tumor marker assays.

The high variability across manufacturers for CA 15-3 was also reported by Slev et al., who performed a comparative analysis of seven automated CA assays and found BE consistently yielding lower results than the SI sub-collective BG (Slev et al., 2006). Similarly, clinical studies have demonstrated considerable method dependent differences for CA 15-3 (Molina et al., 2008), CA 19-9 (Stieber et al., 2008b) and CA 125 (Holdenrieder et al., 2008).

Potential causes of these manufacturer-related differences include the utilization of distinct monoclonal antibodies across assays with different binding sites and affinities due to variable antigen-binding sites, as well as antigen modifications such as glycosylation and different assay formulations (Price et al., 1998; Reinauer and Wood, 2005; Partyka et al., 2012; Zeng et al., 2012; Wojtalewicz et al., 2023). Fortunately, high intra-manufacturer consistency with CVs below 16% was found for all methods studied. This is particularly beneficial when the same methods are applied for monitoring individual patients over time. However, any transition to another method should be carefully managed with double measurements using both methods to minimize disruptions in patient care and ensure continuity in result interpretation. Notably, the low CVs observed here align with similar trends seen in previous marker analyses, such as AFP and CEA, where even lower CVs were observed (Wojtalewicz et al., 2023). Given that certified reference materials (CRM) for AFP and CEA already exist, it is expected that further improvements of CVs for CA 15-3, CA 19-9 and CA 125 will occur once CRMs for these markers are developed (Sturgeon, 2016).

It is important to highlight that the consistent CVs, the high passing rates of the EQA schemes and the considerable differences between the methods remained stable over the studied time interval. A comparison between the present study and an earlier one conducted in 2005 (Reinauer and Wood, 2005) revealed some changes over the past 2 decades. The maximum differences observed were 162% for CA 125, 44% for CA 15%–3% and 195% for CA 19-9. Therefore, manufacturers are urgently called upon to improve the standardization and harmonization of their methods and regulative bodies are encouraged to provide CRMs as a basis for more accurate alignment.

Furthermore, it is imperative that manufacturers conduct clinical performance studies for their tumor marker assays. These studies are essential not only to establish method-specific decision limits for reference intervals in healthy individuals, but also to evaluate criteria for distinguishing between malignant and benign conditions. Additionally, it is crucial to develop criteria for estimating prognosis at different stages of disease and to assess relative increases or decreases in individual patients to measure therapeutic efficacy. This is highly important, as clinical decision criteria will differ for each indication of tumor marker application in cancer patients. Given the considerable variability among individual methods, such studies will enhance the clinical relevance of the assays and optimize their use in patient care.

When differences between methods were related to a normalized median of all methods, a certain bias has to be taken into account, as the RO collective was overrepresented in the whole cohort. Divergent trends in relative medians across individual groups may be attributed to factors such as interfering substances, matrix effects and molecular heterogeneity, particularly for CA 19-9 (Denis et al., 2019; Mahadevarao Premnath and Zubair, 2024). Higher CVs in individual methods can be attributed to interfering substances (Sturgeon and Viljoen, 2011), the simple fact of low participant numbers and variances in assay lot calibration. As reported by Kim et al., the lot effect can result in variances up to 14.3% for CA 19-9 (Kim et al., 2012).

Consequently, the commutability of EQA materials with patient samples is crucial. EQA samples were produced using a human serum-like matrix spiked with the respective tumor antigens from cell cultures. Importantly, the observed manufacturer-specific variations are not necessarily attributable to the spiked material, as similar differences in methods were also observed in plasma samples (van Rossum et al., 2024), with consistently higher concentration of CA 19-9 for AB compared to other manufacturers. Nevertheless, a commutability study with a direct comparison of artificial and patient material is still pending.

Currently, only the EQA acceptance criteria of ±24% for CA 15-3 are defined in the German Medical Association’s RiliBÄK guideline, while criteria for CA 19-9 and CA 125 are not specified (Bundesärztekammer, 2023). Historically, higher acceptance ranges of ±27% for CA 19–9 and ±36% for CA 125 have been defined. These criteria have allowed almost all participants to regularly pass the EQA schemes. However, such broad ranges mean that changes up to 72% for CA 125 might not be interpreted as genuine disease-related changes in individual patients, given the high potential for analytical variability–even when using the same method. Therefore, more stringent limits would be beneficial to enable the clinical interpretation of already smaller dynamic tumor marker changes in individual patients. This approach could help to prevent misdiagnosis and unnecessary invasive tests, as has been discussed in the context of HbA1c measurements (Heinemann et al., 2018).

However, if the limit of ±24% was applied to all three markers, the majority of participants would still pass the EQAs. In contrary, the low variability within methods suggests that even more stringent limits could be feasible. Narrowing the acceptance criteria would improve the quality and reliability of clinical decision-making when interpretating individual tumor marker dynamics. This would be especially relevant for monitoring therapy progress in cancer patients or for disease monitoring after tumor removal. With the new acceptance criteria, changes of 50% could be interpreted reliably. However, this necessitates maintaining consistent methods over longitudinal courses, clearly indicating these methods in laboratory reports and ensuring their inclusion in electronic reports together with the measured values. Furthermore, this information should be incorporated into the newly introduced electronic patient records on a nationwide basis in Germany.

In addition to these measures, manufacturers are encouraged to enhance the standardization and harmonization of tumor marker assays. This includes minimizing manufacturer-specific differences, optimizing assay performance, and conducting clinical studies. Continued collaboration between manufacturers, regulatory agencies, professional organizations, and clinical laboratories is crucial for advancing the field of tumor marker testing and improving the quality of patient care (Aarsand and Sandberg, 2014; Tate et al., 2014; Ceriotti, 2016; Plebani, 2016).

Laboratories within the public health network often encounter challenges during procurement processes, where price considerations may overshadow concerns regarding assay quality and performance. It is crucial to emphasize that tumor marker diagnostics are only valuable if the assays used meet the highest quality standards which should outweigh economic considerations. The results of this longitudinal EQA analysis comparing different methods and manufacturers provide compelling arguments for selecting appropriate assays. These findings may also encourage manufacturers to prioritize assay performance and reliability when developing and calibrating tumor marker assays, thereby enhancing the quality of oncological diagnostics in public health laboratories.

## 5 Conclusion

The present study provides a large set of longitudinal data from EQA schemes for tumor markers CA 15-3, CA 19-9 and CA 125 assessed by different methods and manufacturers. While intra-manufacturer variability was acceptable, inter-manufacturer variability was quite high, which has severe consequences for application of tumor markers in patient care. Therefore, better standardization and harmonization are urgently needed. The development of CRMs and continuous guidance by regulatory bodies will support this process, necessitating close collaboration between manufacturers, regulatory agencies, professional scientific organizations, and clinical laboratories.

Beyond analytical and preanalytical validation, comprehensive clinical studies on the performance of tumor marker tests as well as the definition of meaningful clinical decision criteria for various indications throughout the course of cancer are essential. Improved and internationally aligned acceptance criteria for passing EQA schemes will enable a qualified and sensitive interpretation of longitudinal marker changes in individual cancer patients. These quality indicators are fundamental and should always take precedence over economic consideration. Only through the collaborative efforts of all stakeholders striving for higher quality standards can diagnostic guidance for cancer patients be improved.

## Data Availability

The original contributions presented in the study are included in the article/Supplementary Material, further inquiries can be directed to the corresponding author.
