# Supplementary material for: Longitudinal evaluation of external quality assessment results for CA 15-3, CA 19-9, and CA 125
Source: Front Mol Biosci. 2024 Jun 20;11:1401619. doi: 10.3389/fmolb.2024.1401619 (PMC11222321; doi:10.3389/fmolb.2024.1401619)
Supplement: Supplementary file 3 [file DataSheet1.PDF]

Supplementary Material

This study analyzes longitudinal data for the tumor markers cancer antigen (CA) 15-3 (n=5,492), CA 19-9 (n=6,802), and CA 125 (n=5,362), collected from INSTAND EQAs conducted between 2019 and 2023. We focused on manufacturer collectives with a minimum of six participants per survey, resulting in six collectives for the analysis of the CA 15-3 results, seven collectives for CA 19-9, and six collectives for CA 125. These were, in alphabetical order, Abbott (AB), Beckman (BE), bioMérieux (AX), Diasorin (DO), Roche (RO), Siemens (SI), and Tosoh (TH). Due to the multimodality of the SI collective, we specifically presented the normalized median for the more substantial sub-collectives Bayer Health (BG), DPC Biermann (DG) and Siemens Healthineers (SIE).

1 Supplementary Tables

Supplementary Table 1: CA 15-3, CA 19-9 and CA 125 EQA results between 2019 and 2023.

Supplementary Table 2: Number of participants per manufacturer and total number of participants for the CA 15-3 EQA results from 2019 to 2023.

Supplementary Table 3: Percentage deviation relative to the mean for each manufacturer for CA 15-3, CA 19-9 and CA 125 EQA results between 2019 and 2023.

Please refer to the corresponding Excel tables.

Supplementary Table 4: Comparison of relative, manufacturer-dependent median differences for CA 15-3.

Manufacturer	Min	Max	Median
AB	0.91	1.13	1.03
AX	0.97	1.14	1.05
BE	0.42	0.71	0.54
DO	0.94	1.19	1.06
RO	0.95	1.01	0.98
SI	1.05	1.22	1.12

Supplementary Table 5: Evaluation of manufacturer-dependent CVs for CA 15-3.

Manufacturer	Min [%]	Max [%]	Median [%]
AB	4	11	6
AX	4	16	8
BE	4	14	6
DO	3	14	8
RO	6	16	9
SI	9	23	12
SI – BG	4	13	7
SI – DG	1	16	8
SI – SIE	3	20	7

Supplementary Table 6: Comparison of relative, manufacturer-dependent median differences for CA 19-9.

Manufacturer	Min	Max	Median
AB	2.96	7.61	5.84
AX	1.03	1.98	1.53
BE	1.04	1.92	1.49
DO	1.49	2.61	2.13
RO	0.84	0.96	0.93
SI	1.64	2.80	2.01
TH	0.74	1.29	0.96

Supplementary Table 7: Evaluation of manufacturer-dependent CVs for CA 19-9.

Manufacturer	Min [%]	Max [%]	Median [%]
AB	5	16	8
AX	2	14	5
BE	5	14	8
DO	2	12	7
RO	6	16	9
SI	13	36	25
SI – BG	7	17	10
SI – DG	5	35	12
SI – SIE	4	23	10
TH	3	16	8

Supplementary Table 8: Comparison of relative, manufacturer-dependent median differences for CA 125.

Manufacturer	Min	Max	Median
AB	1.37	1.82	1.54
AX	0.85	1.21	1.06
BE	0.64	1.21	0.79
DO	1.21	1.55	1.44
RO	0.93	0.98	0.96
SI	1.01	1.50	1.26

Supplementary Table 9: Evaluation of manufacturer-dependent CVs for CA 125.

Manufacturer	Min [%]	Max [%]	Median [%]
AB	3	12	5
AX	2	20	8
BE	2	9	5
DO	2	12	5
RO	4	9	6
SI	7	25	18
SI – BG	6	21	8
SI – DG	1	14	6
SI – SIE	2	19	11

2 Supplementary Figures

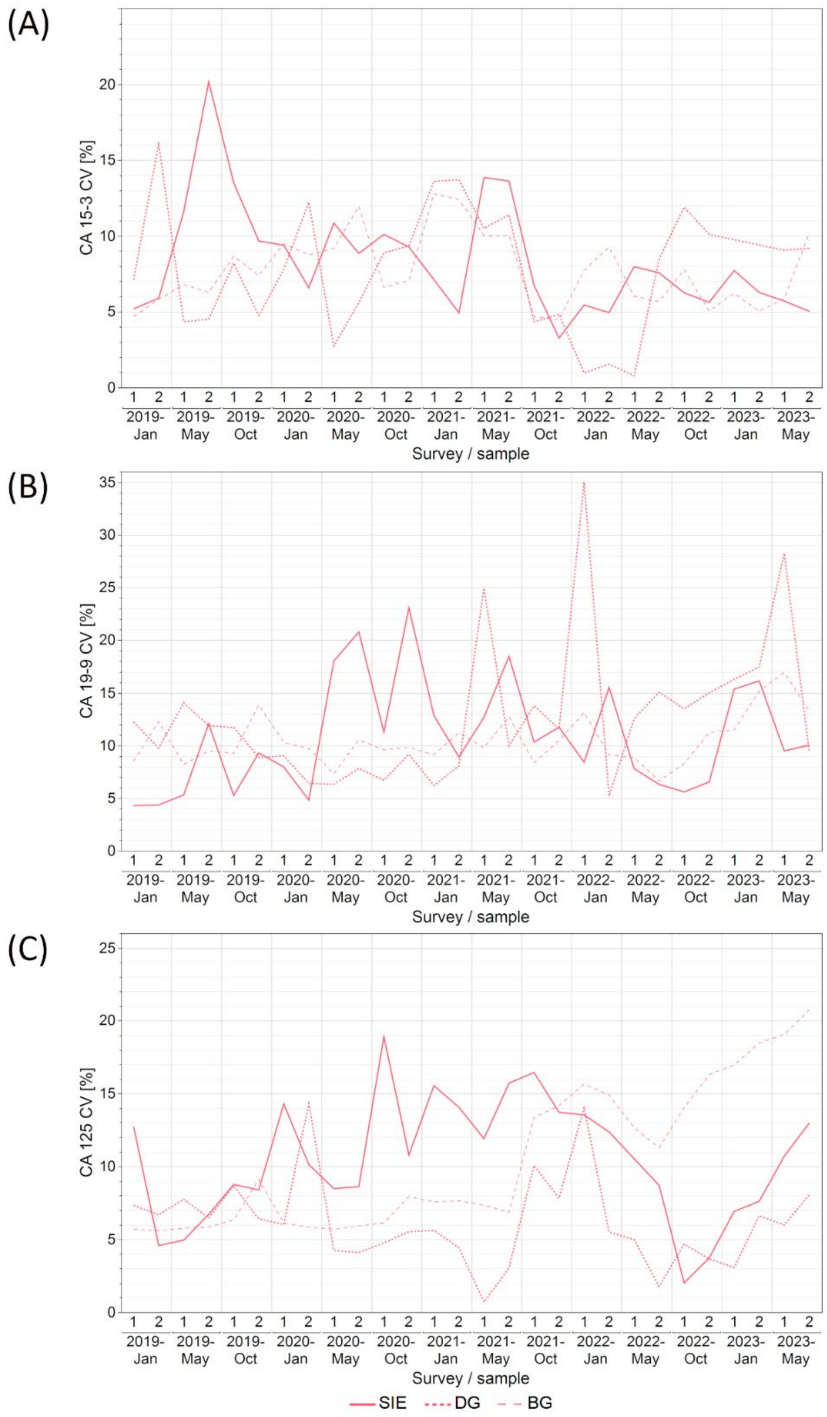

Supplementary Figure 1: Evaluation of manufacturer-dependent CVs between 2019 and 2023 for the SI sub-collectives for CA 15-3 (A), CA 19-9 (B) and CA 125 (C). The three SI sub-collectives are shown as follows: SIE (as a red line), DH (as a red dotted line), and BG (as a dashed line).

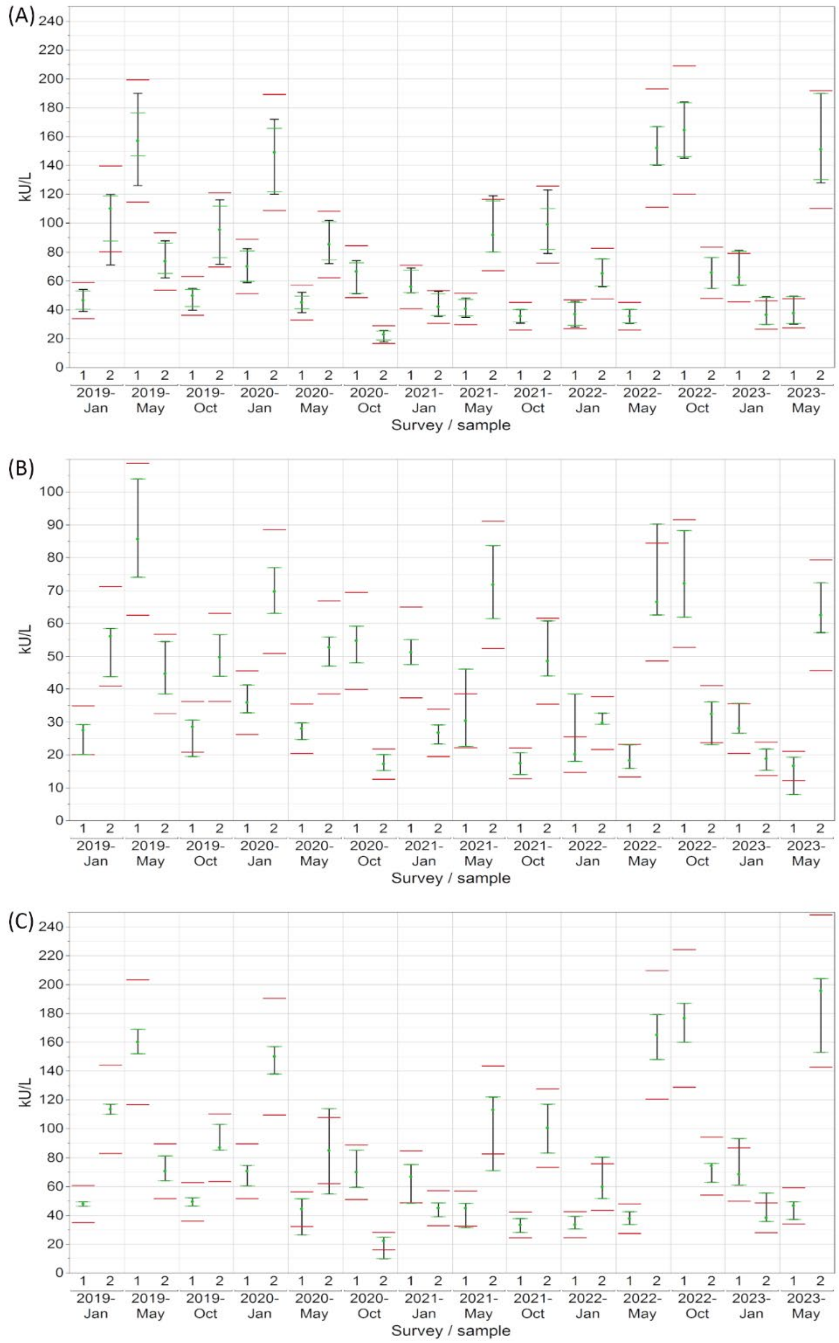

Supplementary Figure 2: Manufacturer-specific evaluation of EQA results for CA 15-3 with respect to the current assessment limits for the SI sub-collectives. The green dot represents the median of all results within each respective collective and EQA survey. Assessment limits of ±36% are highlighted with red lines, while green lines indicate the median for 80% of the results, and a black line signifies the median for 90% of the results.

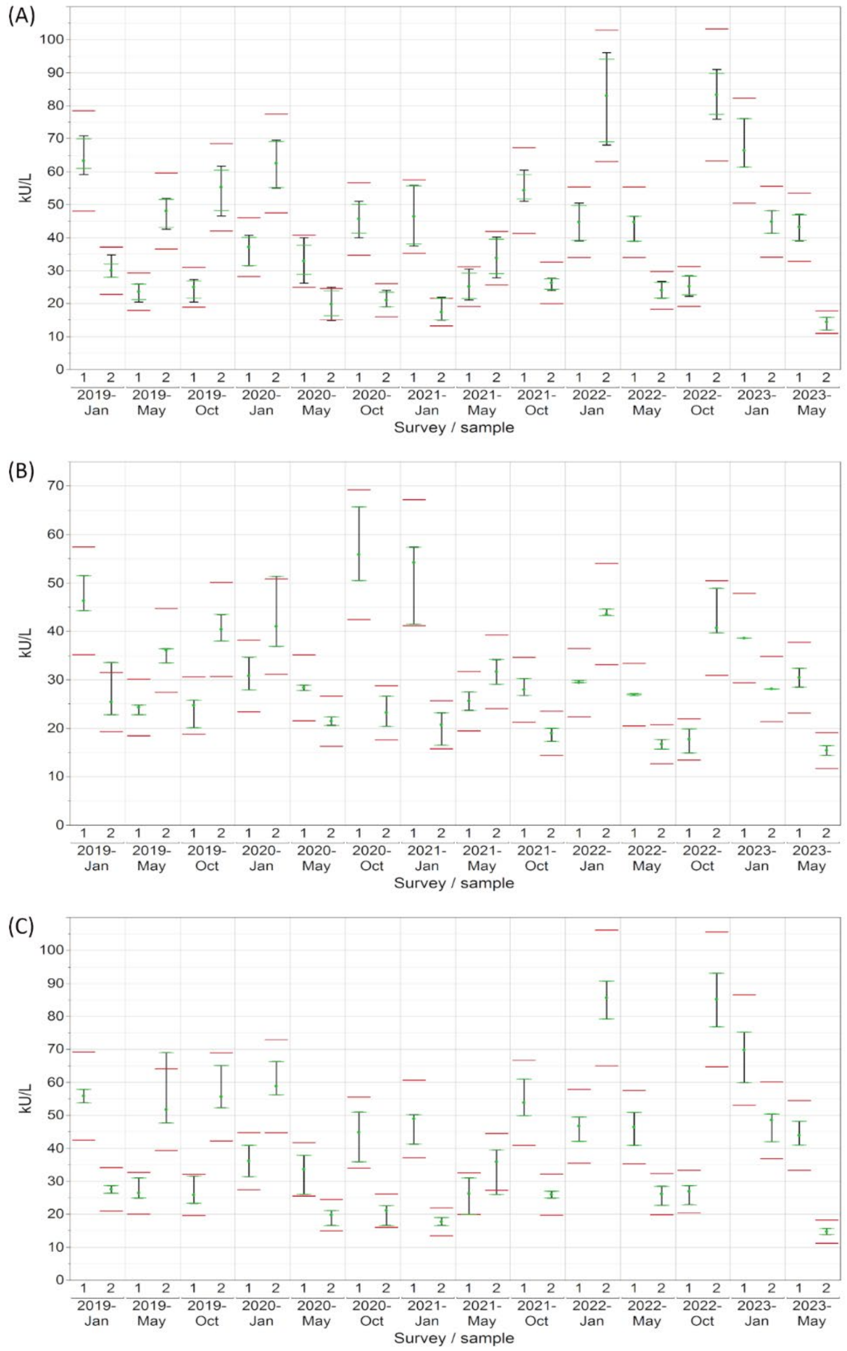

Supplementary Figure 3: Manufacturer-specific evaluation of EQA results for CA 19-9 with respect to the current assessment limits for the SI sub-collectives. The green dot represents the median of all results within each respective collective and EQA survey. Assessment limits of ±36% are highlighted with red lines, while green lines indicate the median for 80% of the results, and a black line signifies the median for 90% of the results.

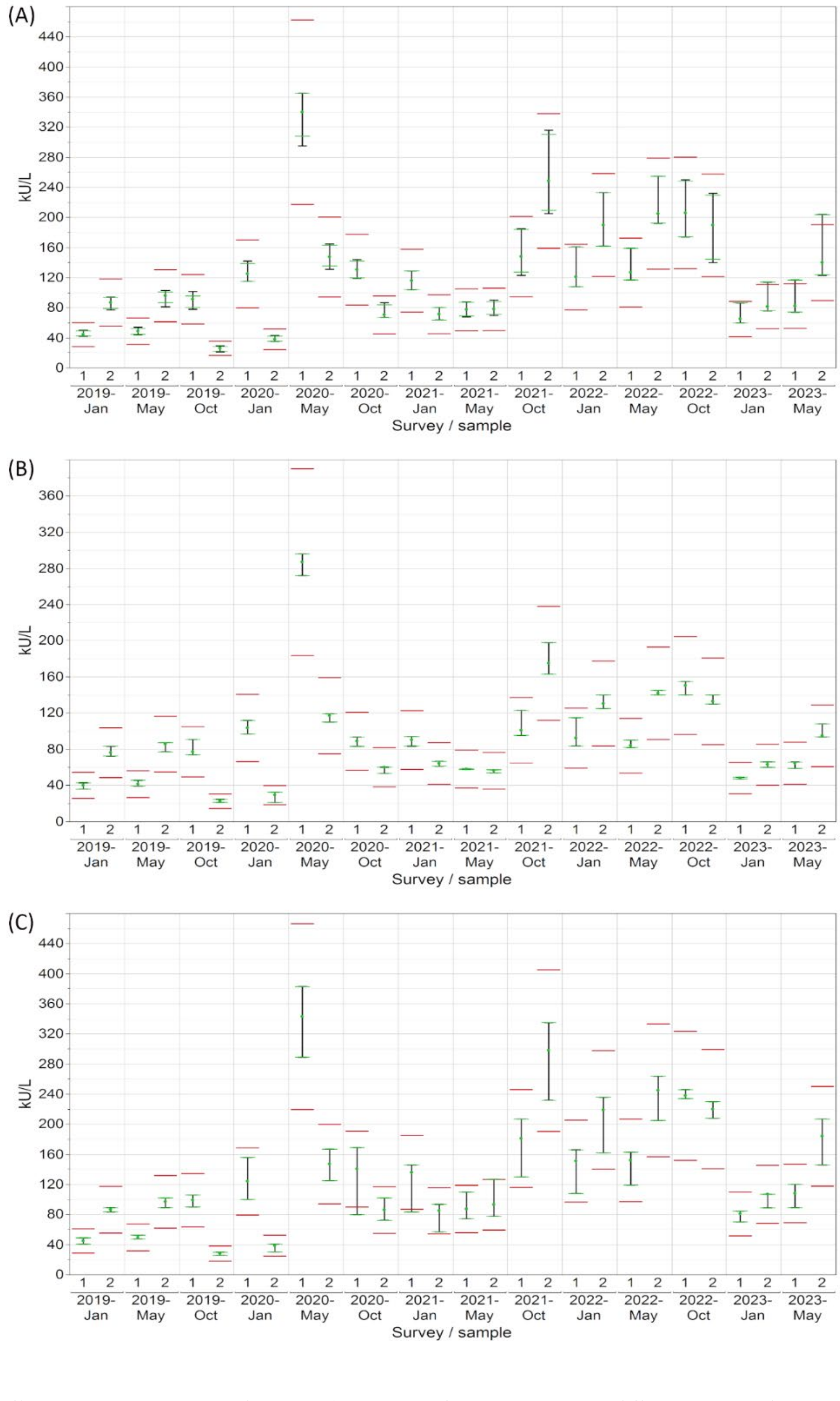

Supplementary Figure 4: Manufacturer-specific evaluation of EQA results for CA 125 with respect to the current assessment limits for the SI sub-collectives. The green dot represents the median of all results within each respective collective and EQA survey. Assessment limits of ±36% are highlighted with red lines, while green lines indicate the median for 80% of the results, and a black line signifies the median for 90% of the results.

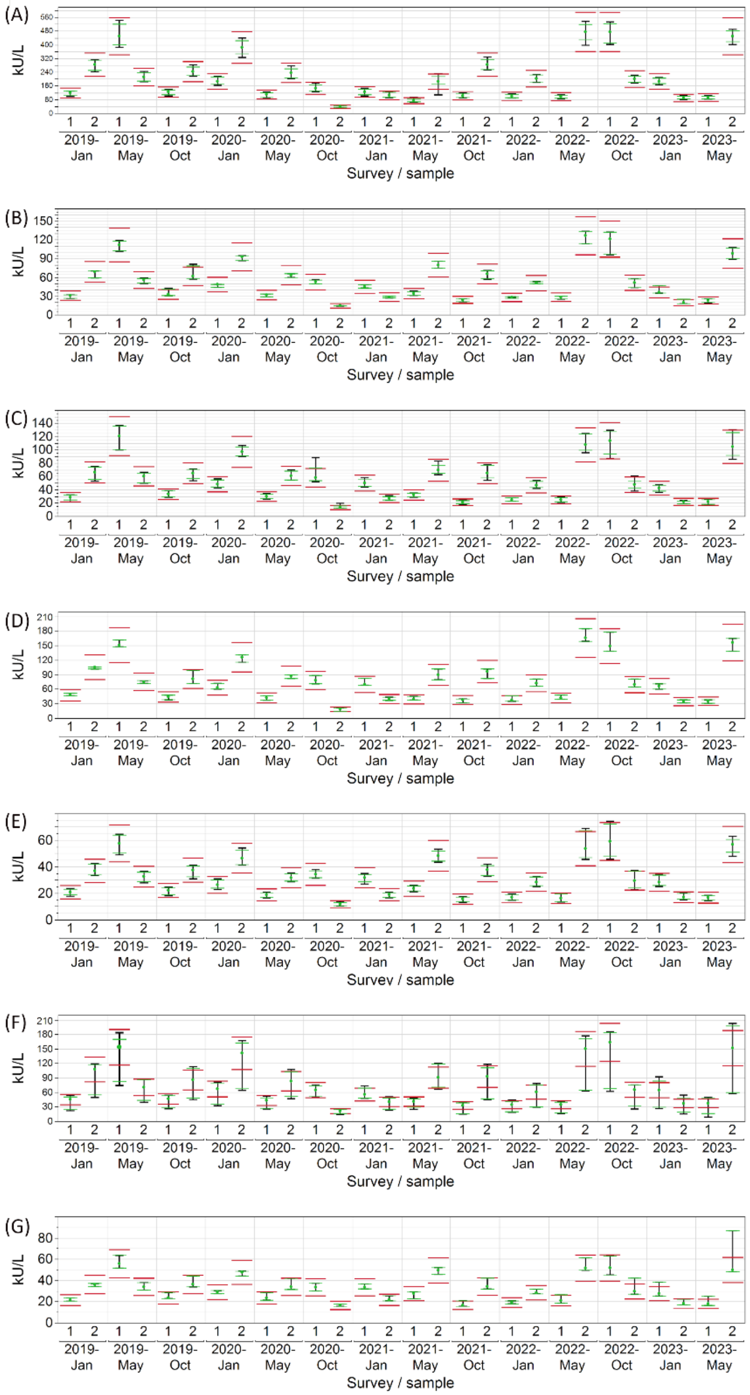

Supplementary Figure 5: Manufacturer-specific evaluation of EQA results for CA 19-9 with respect to the stricter assessment limits of 24% for the AB (A), AX (B), BE (C), DO (D), RO (E), SI (F) and TH (G) collectives. The green dot represents the median of all results within each respective collective and EQA survey. Assessment limits of ±24% are highlighted with red lines, while green lines indicate the median for 80% of the results, and a black line signifies the median for 90% of the results.

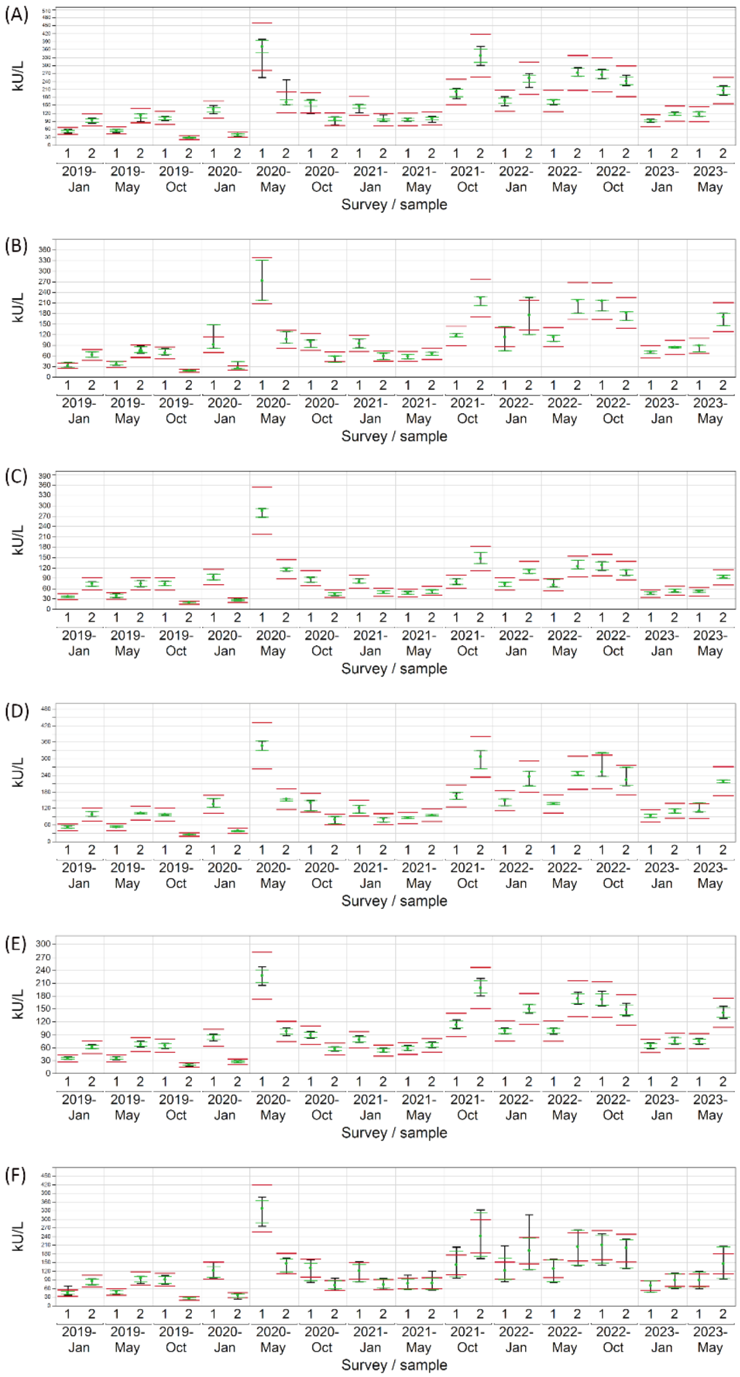

Supplementary Figure 6: Manufacturer-specific evaluation of EQA results for CA 125 with respect to the stricter assessment limits of 24% for the AB (A), AX (B), BE (C), DO (D), RO (E), and SI (F) collectives. The green dot represents the median of all results within each respective collective and EQA survey. Assessment limits of ±24% are highlighted with red lines, while green lines indicate the median for 80% of the results, and a black line signifies the median for 90% of the results.
